# Heterogeneity of T-Tubules in Pig Hearts

**DOI:** 10.1371/journal.pone.0156862

**Published:** 2016-06-09

**Authors:** Hanne C. Gadeberg, Richard C. Bond, Cherrie H. T. Kong, Guillaume P. Chanoit, Raimondo Ascione, Mark B. Cannell, Andrew F. James

**Affiliations:** 1 Cardiovascular Research Laboratories, Bristol Cardiovascular, School of Physiology, Pharmacology & Neuroscience, University of Bristol, Bristol, BS8 1TD, United Kingdom; 2 School of Veterinary Sciences, University of Bristol, Langford House, Langford, BS40 5DU, United Kingdom; 3 School of Clinical Sciences, University of Bristol, Bristol Royal Infirmary, Upper Maudlin Street, Bristol, BS2 8HW, United Kingdom; Scuola Superiore Sant'Anna, ITALY

## Abstract

**Background:**

T-tubules are invaginations of the sarcolemma that play a key role in excitation-contraction coupling in mammalian cardiac myocytes. Although t-tubules were generally considered to be effectively absent in atrial myocytes, recent studies on atrial cells from larger mammals suggest that t-tubules may be more numerous than previously supposed. However, the degree of heterogeneity between cardiomyocytes in the extent of the t-tubule network remains unclear. The aim of the present study was to investigate the t-tubule network of pig atrial myocytes in comparison with ventricular tissue.

**Methods:**

Cardiac tissue was obtained from young female Landrace White pigs (45–75 kg, 5–6 months old). Cardiomyocytes were isolated by arterial perfusion with a collagenase-containing solution. Ca^2+^ transients were examined in field-stimulated isolated cells loaded with fluo-4-AM. Membranes of isolated cells were visualized using di-8-ANEPPS. T-tubules were visualized in fixed-frozen tissue sections stained with Alexa-Fluor 488-conjugated WGA. Binary images were obtained by application of a threshold and t-tubule density (TTD) calculated. A distance mapping approach was used to calculate half-distance to nearest t-tubule (*HD*_*TT*_).

**Results & Conclusion:**

The spatio-temporal properties of the Ca^2+^ transient appeared to be consistent with the absence of functional t-tubules in isolated atrial myocytes. However, t-tubules could be identified in a sub-population of atrial cells in frozen sections. While all ventricular myocytes had TTD >3% (mean TTD = 6.94±0.395%, *n* = 24), this was true of just 5/22 atrial cells. Mean atrial TTD (2.35±0.457%, *n* = 22) was lower than ventricular TTD (P<0.0001). TTD correlated with cell-width (r = 0.7756, *n* = 46, P<0.0001). *HD*_*TT*_ was significantly greater in the atrial cells with TTD ≤3% (2.29±0.16 μm, *n* = 17) than in either ventricular cells (1.33±0.05 μm, *n* = 24, P<0.0001) or in atrial cells with TTD >3% (1.65±0.06 μm, *n* = 5, P<0.05). These data demonstrate considerable heterogeneity between pig cardiomyocytes in the extent of t-tubule network, which correlated with cell size.

## Introduction

The transverse tubular network (t-tubules) is formed from invaginations of the sarcolemma and plays a key role in excitation-contraction (EC) coupling in mammalian cardiac ventricular myocytes [1, 2]. In ventricular myocytes, the t-tubules form part of a complex rete network closely associated with z-lines [3]. The L-type Ca^2+^ channels in the t-tubular membrane activate clusters of RyR in the closely juxtaposed junctional sarcoplasmic reticulum (SR) membrane, ensuring the efficient coupling of Ca^2+^ entry to Ca^2+^ release and a co-ordinated release of Ca^2+^ in the cell [1, 2, 4]. In ventricular cells, disconnection of t-tubules from the sarcolemma (by osmotic shock) results in marked spatiotemporal abnormalities in the ventricular Ca^2+^ transient such that Ca^2+^ release is initiated at the cell edge and propagates centripetally by diffusion. As a result, the peak of the transient at the cell center has lower amplitude and is delayed relative to the transient at the cell edge [5, 6]. Moreover, in heart failure, disruption of the t-tubule network impairs the efficiency of coupling between Ca^2+^ influx and CICR, resulting in a slowed and dyssynchronous release of Ca^2+^, which is suggested to contribute to the contractile dysfunction [7–9]. Disruption to t-tubule function may also contribute to arrhythmogenesis [10].

In contrast to ventricular myocytes, the role of t-tubules in atrial myocytes is less clear. The sparsity of the t-tubular network in atrial myocytes in smaller mammalian species (e.g. cat, guinea pig, mouse, rabbit, rat) [11–18] leads to a Ca^2+^ transient that initiates at the periphery of the cell and propagates towards the cell center, reminiscent of the spatiotemporal properties of detubulated ventricular myocytes [19–25] and cardiac Purkinje cells lacking t-tubules [26].

It has long been considered that the human atrium lacked t-tubules [27]. However, more recent studies have demonstrated the existence of some t-tubules in the atria of larger species (i.e. dog, cow, horse, sheep, pig), including human [28–32]. It has also been suggested that the limited atrial t-tubular network may be disrupted in sheep models of AF and heart failure [29, 30] and that abnormalities in the existing t-tubule network may contribute to atrial contractile dysfunction and arrhythmogenesis in cardiac disease [33]. The pig has been suggested to represent a suitable large animal model for translational studies of human health and disease and the existence of t-tubules in the atrial of pig hearts has recently been demonstrated [32, 34]. The objective of the present study was to examine the degree of heterogeneity in the extent of t-tubules in pig atria in comparison with ventricular tissue from normal pig hearts.

## Methods

### Pig heart tissue

All procedures were approved by University of Bristol Research Ethics committee and performed in accordance with the Guide for the Care and Use of Laboratory Animals [35] and the United Kingdom Animal (Scientific Procedures) Act, 1986, under Home Office project licence PPL 30/2854. Young adult female Landrace White pigs (45–75 kg, 5–6 months of age) from sham/control group were subject to general anesthesia (pre-medication with ketamine, i.m. 15–20 mg/kg, induction with propofol i.v. 16–20 mg/kg and maintained with isoflurane). The study was restricted to female pigs in order to limit the possible contribution of sex differences in cardiac structure and function to the heterogeneity between animals. Control animals were subject to median sternotomy with no further intervention while Sham animals were subject to median sternotomy followed by cardiopulmonary bypass with cardioplegia arrest. At the termination of the procedure, the hearts were removed and transported to the laboratory in chilled cardioplegic solution containing (in mM): 50 KH_2_PO_4_, 8 MgSO_4_, 10 HEPES, 5 adenosine, 140 D-glucose, 100 mannitol, pH to 7.4 with KOH.

In the laboratory, in preparation for either cell isolation or perfusion fixation, a wedge of tissue from the back of the heart incorporating the left atrial posterior free wall and the base of the left ventricular free wall was removed from the heart by cutting through the left ventricular wall around the base of the atrium and up through the aorta. The circumflex branch of the left coronary artery was cannulated and perfused with Tyrode’s solution. Any leaking branches of the circumflex artery were tied off to enable perfusion of the tissue wedge.

### Pig myocyte isolation

Myocytes from either the left atrium or the left ventricle were isolated by coronary perfusion of the tissue, as described above, with a collagenase-containing Tyrode’s solution. The tissue was initially perfused with calcium-free solution containing (in mM): 137 NaCl, 5 KH_2_PO_4_, 1 MgSO_4_, 5 HEPES, 10 D-glucose, 10 taurine, pH 7.4 with NaOH for 5 min before switching to EGTA solution (calcium-free solution plus 200 μM Na-EGTA) for 5 min. The tissue wedge was then perfused with enzyme-containing solution (calcium-free solution plus 1 U/ml type I collagenase (Worthington), 3 U/ml type XXIV protease (Sigma) and 240 μM CaCl_2_) until the tissue became soft. Generally, this was achieved after approximately 15 min of perfusion. Small pieces of tissue were selected from the perfused area of either the left atrium or the left ventricle and placed in ‘Kraftbrühe’ (KB) solution containing (in mM) 100 L-glutamic acid, 30 KCl, 10 HEPES, 1 EGTA, 5 Na-pyruvate, 20 taurine, 10 D-glucose, 5 MgCl_2_, 5 succinic acid, 5 creatine, 5 adenosine 5’-triphosphate disodium salt, 5 β-OH butyric acid, pH 7.2 with KOH [36]. Tissue chunks were then gently triturated until the solution became cloudy reflecting dissociation of cells. Excess pieces of tissue were removed and cells stored at 4°C until use.

### Intracellular calcium measurements

Isolated cells (1 ml in KB solution) were loaded with 5 μM Fluo-4-AM (5 μl 1 mM stock in 2.5% Pluronic® F-127 in dimethylsulfoxide into 1 ml cell suspension) for 20 min. The dye was removed by centrifugation of the cells at approximately ×100 g for 30 s and aspiration of the supernatant. Cells were re-suspended in Tyrode’s solution and left to de-esterify for 20 min before use. Once settled onto a cover slip based chamber on the stage of a confocal microscope, cells were superfused with Tyrode’s solution containing (in mM): 140 NaCl, 4 KCl, 5 HEPES, 10 glucose, 1 CaCl_2_, 1 MgCl_2_, pH 7.4 with NaOH and stimulated at 1 Hz via platinum electrodes in the bath. Myocytes were visualized with a Zeiss Pascal LSM5 laser-scanning confocal microscope with the aperture set to 1 Airy Unit. Fluo-4 was excited at 488 nm and light collected at wavelengths greater than 505 nm. ‘Linescan’ images were obtained by repeatedly scanning transversely across the cell at the same location once every 1.92 ms. All experiments were carried out at room temperature (RT).

### Tissue freezing and sectioning

Tissue wedges dissected from the left atrial posterior free wall or the left ventricular free wall near the base were perfused with phosphate-buffered saline (PBS; GIBCO, Life Technologies Ltd., Paisley, UK) for 5 min and then with 4% neutral buffered formalin in PBS. Small samples of tissue (approximately 1 mm^3^) were dissected from either the left atrial posterior free wall or the left ventricular free wall at the base of the heart and fixation completed in 4% neutral buffered formalin for 1 hour at 4°C, before cryo-protection with sequential 10%, 20% and 30% sucrose in PBS (1 hour each at 4°C). Tissue was frozen in liquid N_2_-cooled isopentane and stored at -80°C until sectioning. Tissue was sectioned using a cryostat after embedding in Tissue-Tek® and sections of either 10 μm or 40 μm thickness cut. Tissue sections were collected on poly-l-lysine coated microscope slides and kept at -20°C before staining.

### Membrane staining

The surface sarcolemma including t-tubules of isolated myocytes were stained by incubating in di-8-ANEPPS at 5 μM for 5 min, then pelleted by centrifugation at approximately ×100 g and re-suspended in Tyrode’s solution. Cells were visualized on a Zeiss Pascal LSM5 laser scanning confocal microscope using a 1.4 NA 63× objective lens with the pinhole set to 1 Airy unit. Di-8-ANEPPS was excited at 488 nm and light collected at wavelengths greater than 505 nm.

Tissue slices were stained with Alexa-Fluor® 488-conjugated wheat-germ agglutinin (WGA) and Alexa-Fluor® 633-conjugated phalloidin. Microscope slides were allowed to warm to RT before adding 5 μg/ml WGA over the tissue sections. WGA was left for 1 h at RT before washing in PBS. Tissue was then stained with phalloidin at 5 U/ml for 1 h at RT before washing again and leaving to air dry. Slides were mounted with Vectashield® containing DAPI and sealed with nail polish. Tissue slices were visualized on a Leica SP5 confocal imaging system using a 1.4 NA 63× objective lens equipped with 405, 488 and 633 nm lasers with the confocal aperture set to 1 Airy unit. DAPI, Alexa-Fluor® 488 and 633 emissions were detected between 400–480, 500–550 and 650–700 nm, respectively.

### Image analysis

Cell and tissue images were analyzed using a combination of IDL 8.2 (Exelis VIS UK, Bracknell, UK), MATLAB (Mathworks UK, Cambridge, UK), FIJI, Microsoft Excel and Prism (vs 5, GraphPad Software Inc. La Jolla, CA, USA). Images were deconvolved in IDL using the Richardson-Lucy algorithm with a 3D Gaussian point spread function calculated from the measured full-width at half maximum in x, y and z of 0.17 μm yellow-green Fluospheres (Life Technologies Ltd., Paisley, UK) [37].

Di-8-ANEPPS staining was quantified from the power spectrum calculated from the 2D FFT of each of the central 3 slices of a z-series through the cell. The power spectrum was normalized to the amplitude of the frequency-independent component. The amplitude of the first harmonic represents an index of t-tubule regularity, often referred to as ‘t-tubule power’ [38].

Single cells were selected from the WGA/phalloidin-stained tissue sections by drawing a selection around the cell and then by creating a mask from this selection to exclude neighboring cells. Cells with clear surface membrane staining evident in at least 5 optical sections were selected for analysis. WGA staining of the nuclei and Golgi apparatus [39, 40] was removed from measurements by constructing a binary mask based on DAPI fluorescence and the perinuclear Golgi. Images of single cells in 3-D were constructed from a z-series of images using FIJI and the short axis diameter of each cell was measured. WGA-stained images were binarized with a threshold set to 1.5 times the mean pixel intensity of the whole cell selection. The edge of the cell at the surface sarcolemma (SS) was detected using a gradient vector flow (GVF) algorithm [41] and the edge of the cell defined as a boundary within 3 pixels of the edge. T-tubules were defined as regions within this boundary that were > 1.5 times the mean pixel intensity of the whole cell selection. From these masked images, Euclidian distance maps were calculated and cumulative distributions of values for the surface sarcolemma (SS), all cell membranes (WC), including the SS and t-tubules (TT) were derived to allow half-distances to the nearest membrane (*HD*_*WC*_, *HD*_*TT*_ and *HD*_*SS*_) to be calculated. Since there were no significant differences in the t-tubule network between Sham (3 hearts; atrial cells *n* = 20, ventricular cells *n* = 15) or control (4 hearts; atrial cells *n* = 12, ventricular cells *n* = 9), data from sham and control animals were pooled.

### Modelling

To illustrate the effect of cell diameter on diffusion of Ca^2+^ to the center of the cell, a simple cylindrical diffusion model was used with a single fast fixed buffer. We considered cylindrical geometry to be an adequate first order approximation for the cell which allowed the diffusion problem to be discretized in one dimension with 99 computational elements. The reaction/diffusion equation was coded and solved with the Facsimile program (see [42]). The free Ca^2+^ diffusion coefficient was set to 3.5 x 10^−10^ dm^2^/s to simulate the presence of an instantaneous Ca^2+^ buffer with a buffering power of 100. This approximation proved adequate to reproduce the time course of Ca^2+^ rise in small diameter atrial cells (r = 5.13 μm) lacking t-tubules which then allowed estimation of the delay for Ca^2+^ rise in larger cells that would occur in the absence of t-tubules which synchronize release [43].

### Statistics

All data were subject to a D’Agostino Pearson normality test. Sample sizes are reported as the number of hearts (*N*) and the number of cells (*n*). Data are presented as mean ± standard error of the mean (SEM). The effect of section orientation on TTD and *HD*_*TT*_ were compared by Student’s unpaired t-test. Short axis length and TTD were compared between groups by one-way ANOVA with Bonferroni post hoc test and half-distances were compared by repeated measures two-way ANOVA and Bonferroni multiple comparisons test. Spearman’s correlation coefficient was calculated for the correlation between t-tubule density and short axis diameter. P<0.05 was used as the acceptable limit of statistical confidence.

## Results

**Fig 1:**
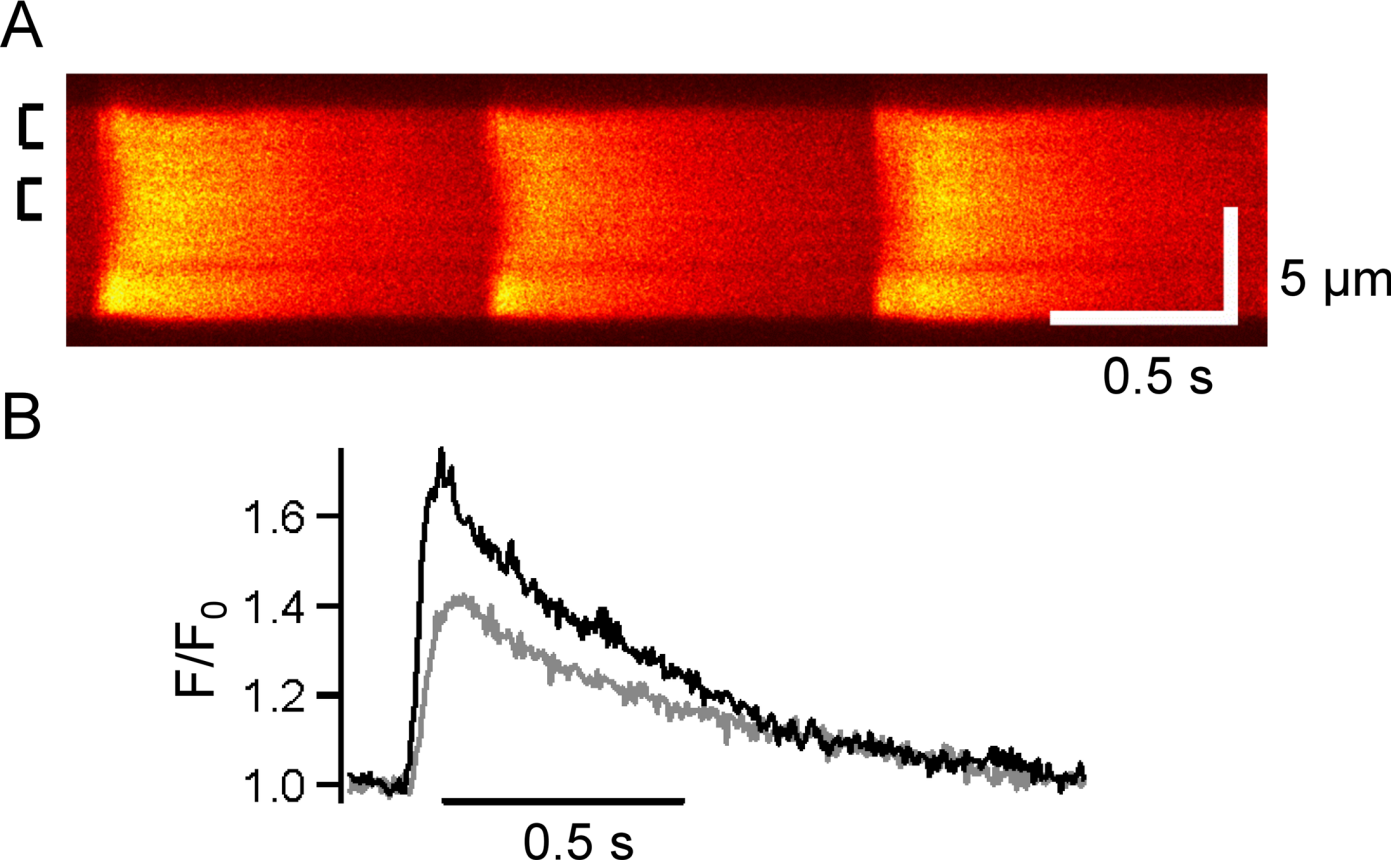
Spatiotemporal properties the atrial Ca^2+^ transient. A) Representative linescan of a fluo-4-loaded pig isolated atrial myocyte. Vertical scale represents 5 μm. Boxes on the left indicate regions averaged to produce traces shown in (B). B) Normalized fluorescence (F/F_0_) of averaged transients at the edge (black) and center (grey) of the cell. Global diastolic level between transients was used as F_0_. Data are representative of 4 cells from 2 pigs.

Isolated atrial myocytes field-stimulated at 1 Hz had Ca^2+^ transients that were not uniform, with Ca release first appearing at the cell edge and spreading with a delay toward the center, where a smaller amplitude transient occurred (Fig 1). These data are consistent with the initiation of Ca^2+^ release at the periphery of the cell which then propagated centripetally, as has been reported in atrial cells lacking t-tubules from smaller mammalian species (i.e. cat, guinea pig, mouse, rabbit and rat) [11–18] as well as Purkinje fiber cells [26].

**Fig 2:**
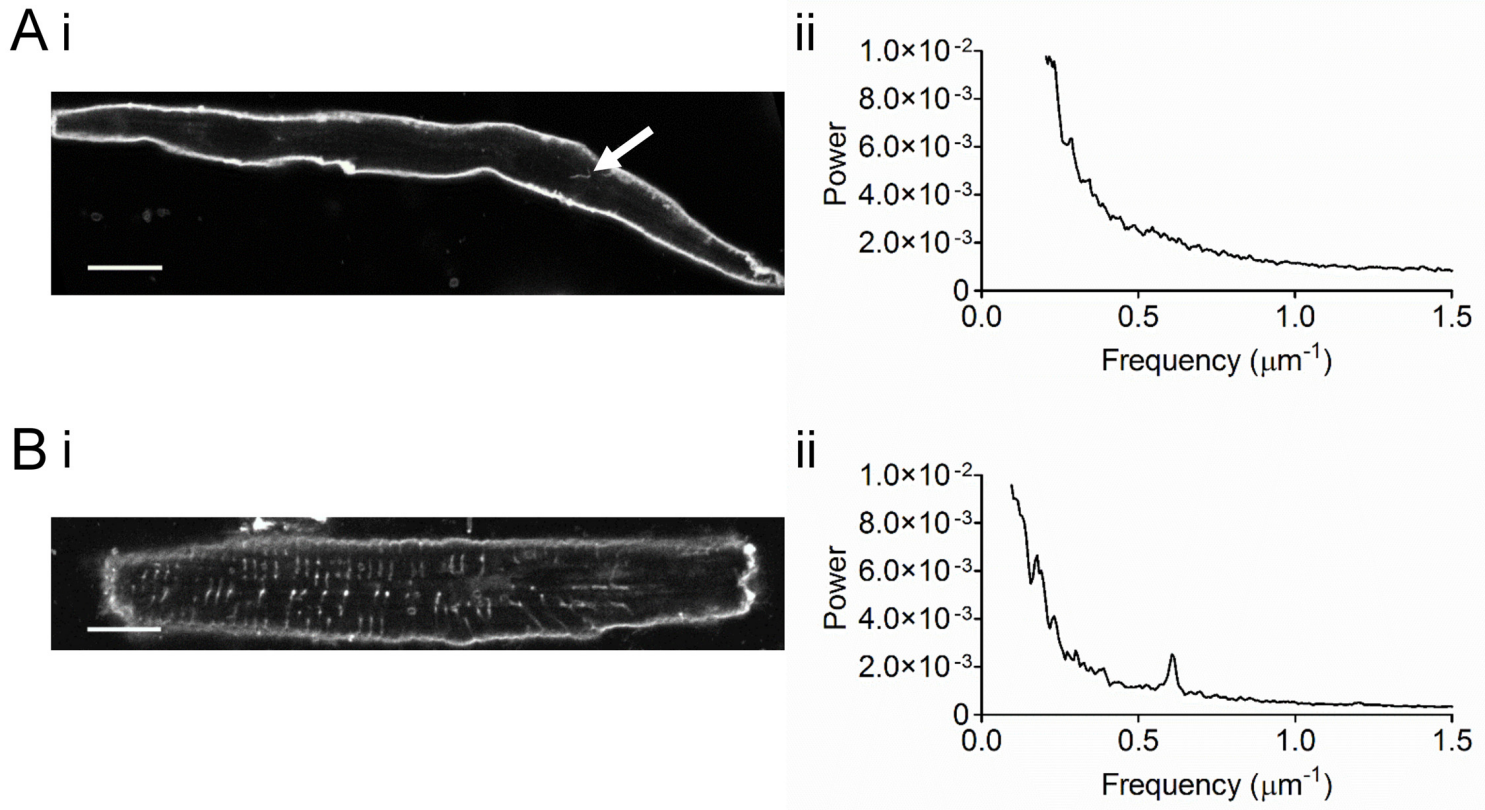
Di-8-ANEPPS-stained isolated atrial and ventricular myocytes. A) Representative image of a di-8-ANEPPS-stained isolated atrial myocyte (i) and corresponding power spectrum (ii). Data are representative of 10 cells from 3 pigs. B) Representative image of a di-8-ANEPPS-stained isolated ventricular myocyte (i) and corresponding power spectrum (ii). Arrow indicates staining of t-tubule network. Scale bars represent 12 μm. Data are representative of 4 cells from 1 pig. The mean (±SEM) of the frequency corresponding to the peak was 0.588±0.004 μm^-1^ and the mean amplitude was 2.45×10^−3^±7.12×10^−4^ (*n* = 4).

The occurrence of t-tubules was examined in *isolated* atrial and ventricular myocytes stained with Di-8-ANEPPS (Fig 2). Membrane staining of pig atrial cells was generally restricted to the cell edge without any obvious transverse pattern (Fig 2Ai), quite unlike ventricular myocytes which showed a striated pattern of staining consistent with the presence of t-tubules at the z-line (Fig 2Bi). Nevertheless, it was possible to detect occasional small tubular elements in cells (see arrows in Fig 2Ai). The relative lack and irregularity of t-tubules is underscored by the power spectra of these cells: Ventricular cells showed regular staining with a peak to the power spectrum corresponding to a mean sarcomere length of 1.70 μm (Fig 2Bii) while no such peak was seen in atrial cells (Fig 2Aii). Taken together, these data suggest that atrial cells isolated from pig hearts possess a very limited t-tubule network.

**Fig 3:**
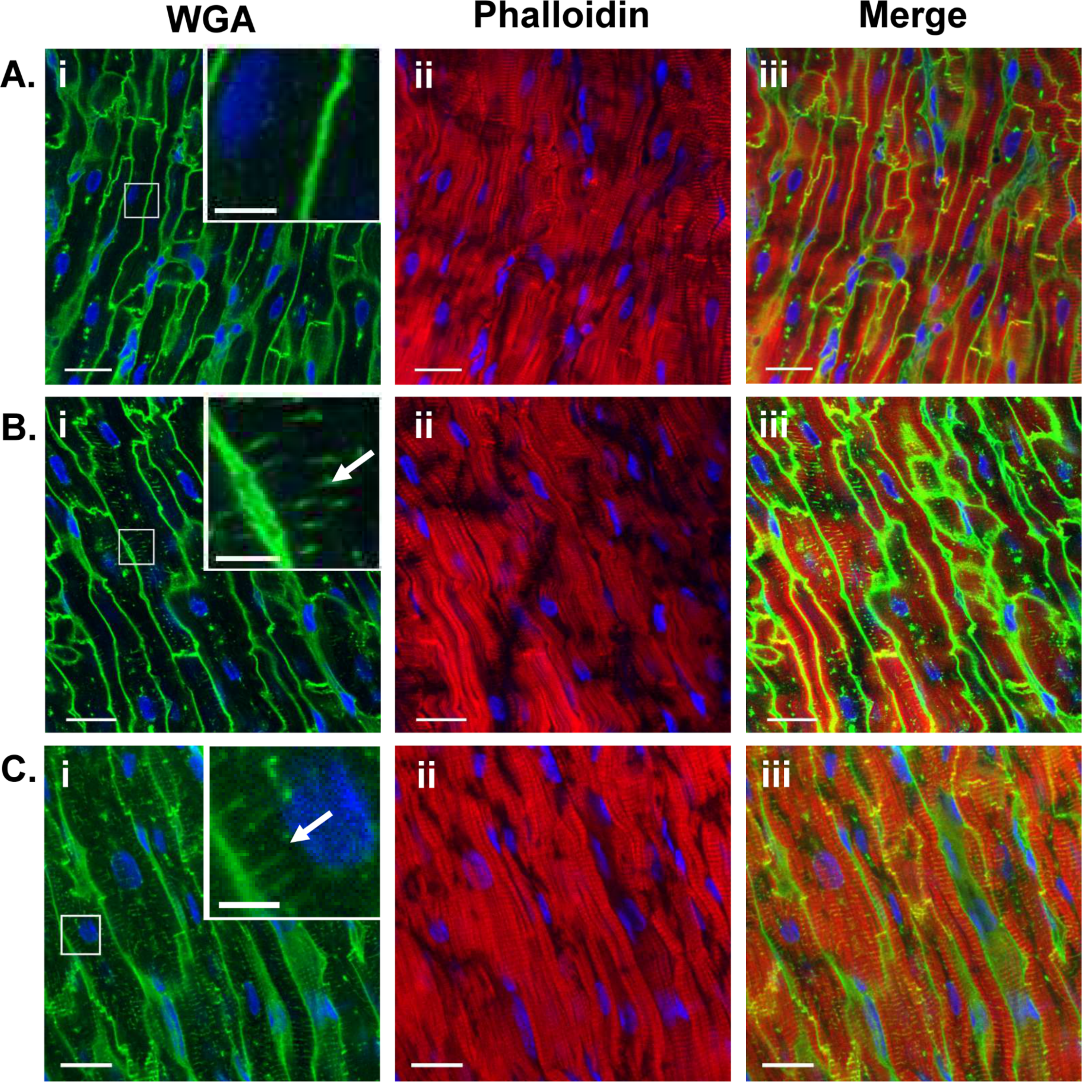
T-tubules in fixed sections of pig atrial and ventricular tissue. Representative examples in the longitudinal plane. Pig atrial tissue without extensive t-tubules (A), pig atrial tissue with extensive t-tubules (B) and ventricular tissue (C) stained with Alexa Fluor-488 WGA (green; i) and Alexa Fluor-633 phalloidin (red; ii). Overlay shown in (iii). Inserts in (i) show detail at a higher magnification. DAPI staining shown in blue in all images. Scale bars represent 17 μm.

However, it is known that the t-tubular system is labile [44] and there is the possibility that a fragile t-tubular system might be lost during cell isolation. Therefore tissue sections were also labelled with WGA and phalloidin to visualize membrane and actin filaments respectively and examples are shown in Fig 3.

The majority of pig atrial cells (17/22) showed WGA staining predominantly around the cell periphery (Fig 3Ai) while staining in pig ventricular tissue also occurred as thin elements generally radially directed towards the cell center, consistent with an extensive t-tubule structure similar to human [45] and unlike rat [3], which has many more circumferential elements giving the appearance of a rete (Fig 3Ci). However, the presence of a limited t-tubule network was also detected in a minority (5/22) of atrial sections (Fig 3Bi).

**Fig 4:**
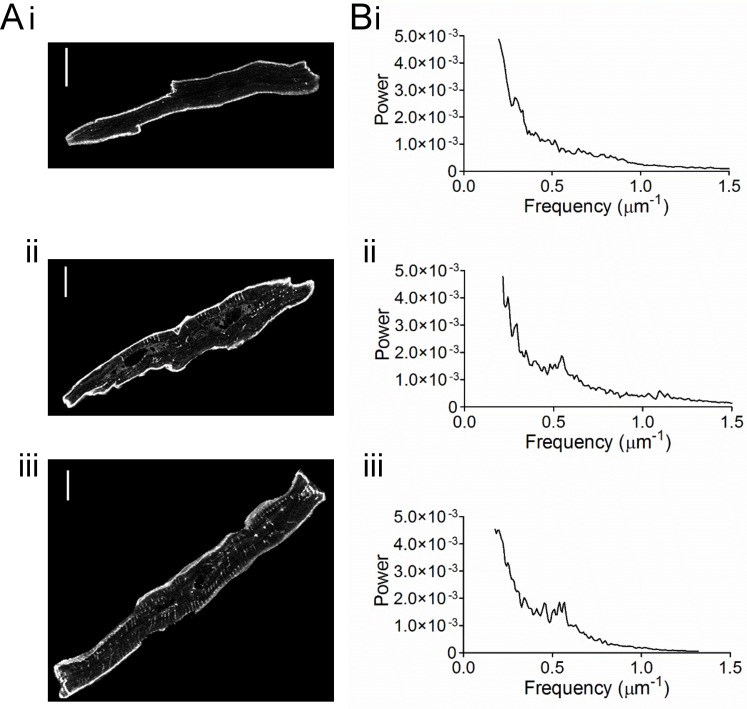
T-tubule density in atrial and ventricular cells from stained tissue sections. A) Selected representative examples of single (i) atrial cells without t-tubules, (ii) atrial cells with t-tubules and (iii) ventricular cells. Scale bars represent 12 μm. TTD and short axis diameter were, respectively, 1.8502 and 11.73 μm (i), 7.7678 and 22.14 μm (ii) and 7.7414 and 20.05 μm (iii). B) Corresponding power spectra for the cells shown in A. The peaks in Bii and Biii were at, respectively, 0.535 and 0.539 μm^-1^.

Individual cells were identified in images and selected for the calculation of TTD and distance mapping (Fig 4). In 17 atrial myocytes that showed a limited t-tubule network, t-tubule labelling occupied 1.305±0.171% of the cell cytoplasm (Fig 4Ai). In comparison, the well-developed t-tubule network staining of ventricular myocytes occupied 6.945±0.395% of the cell cytoplasm (Fig 4Aii) (*n* = 24) which was highly significant (P<0.0001). Nevertheless, there was overlap between the TTD distributions in the two cell types with a few atrial cells having a t-tubule density approaching that of ventricular cells.

**Fig 5:**
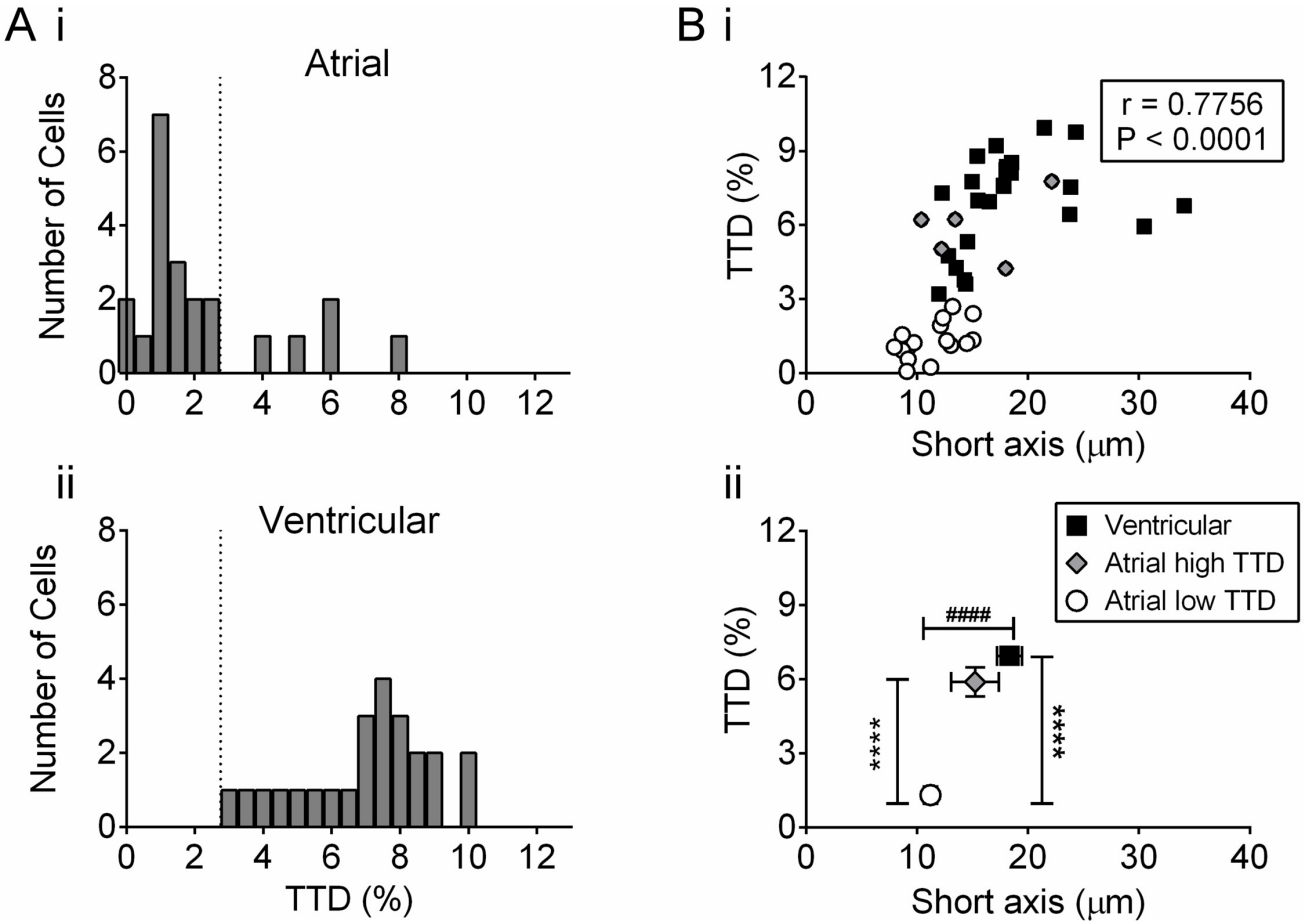
Distribution of t-tubule density in atrial and ventricular cells from tissue sections. A) Frequency distributions of, respectively, atrial (i) and ventricular (ii) myocyte t-tubule density. B) Correlation of t-tubule density with short axis diameter. (i) Data shown separately for atrial cells with ‘low TTD’ (open circles, *n* = 17), in atrial cells with ‘high TTD’ (grey-filled diamonds, *n* = 5) and in ventricular cells (black-filled squares, *n* = 24). Spearman’s correlation coefficient is shown (*n* = 46). TTD was correlated with short axis diameter for both atrial cells (r = 0.6025, P = 0.0030, *n* = 22) and ventricular cells (r = 0.4887, P = 0.0154, *n* = 24). (ii) Data shown as mean±SEM for each cell type. ** P<0.01, *** P<0.0001 one-way ANOVA with Bonferroni post-test for TTD. ## P<0.01, ### P<0.0001 one-way ANOVA with Bonferroni post-test for short axis diameter. Data were obtained from atrial and ventricular sections obtained from 3 pigs for either cell type.

During these experiments we noticed that t-tubules were seen more frequently in larger cells, regardless of whether these were atrial or ventricular cells. Therefore, the hypothesis that the existence of t-tubules is related to cell size was tested. TTD significantly correlated (P<0.0001) with the short axis diameter in the combined group of all 46 cells selected, consistent with cell size being a factor in determining the extent of the t-tubule network (Fig 5Bi). Atrial cells were divided into two sub-groups; those with TTD ≤ 3% (‘low TTD’) and the other with TTD > 3% (‘high TTD’), based on the observed lower limit of ventricular TTD density (dotted lines, Fig 5A). Atrial cells with high TTD did not differ from ventricular cells in either mean TTD or short axis diameter (Fig 5Bii). On the other hand, atrial cells with low TTD were significantly smaller than both atrial cells with high TTD and ventricular cells, consistent with cell size being an important factor in determining t-tubule density (Fig 5Bii). The orientation of sections did not affect either the mean half-distance to TT membrane (*HD*_*TT*_) or TTD (transverse section *HD*_*TT*_, 1.27±0.06 μm, *n* = 12; oblique section 1.40±0.07 μm, *n* = 12; transverse section TTD, 7.31±0.42%; oblique section, 6.58±0.67%) in ventricular cells. Similarly, in atrial cells with TTD < 3%, neither mean *HD*_*TT*_ (longitudinal section, 2.73±0.22 μm, *n* = 5; oblique section, 2.10±0.19 μm, *n* = 11) nor TTD (longitudinal section, 1.39±0.37%; oblique section, 1.30±0.21%) were significantly affected by the orientation of the sections.

**Fig 6:**
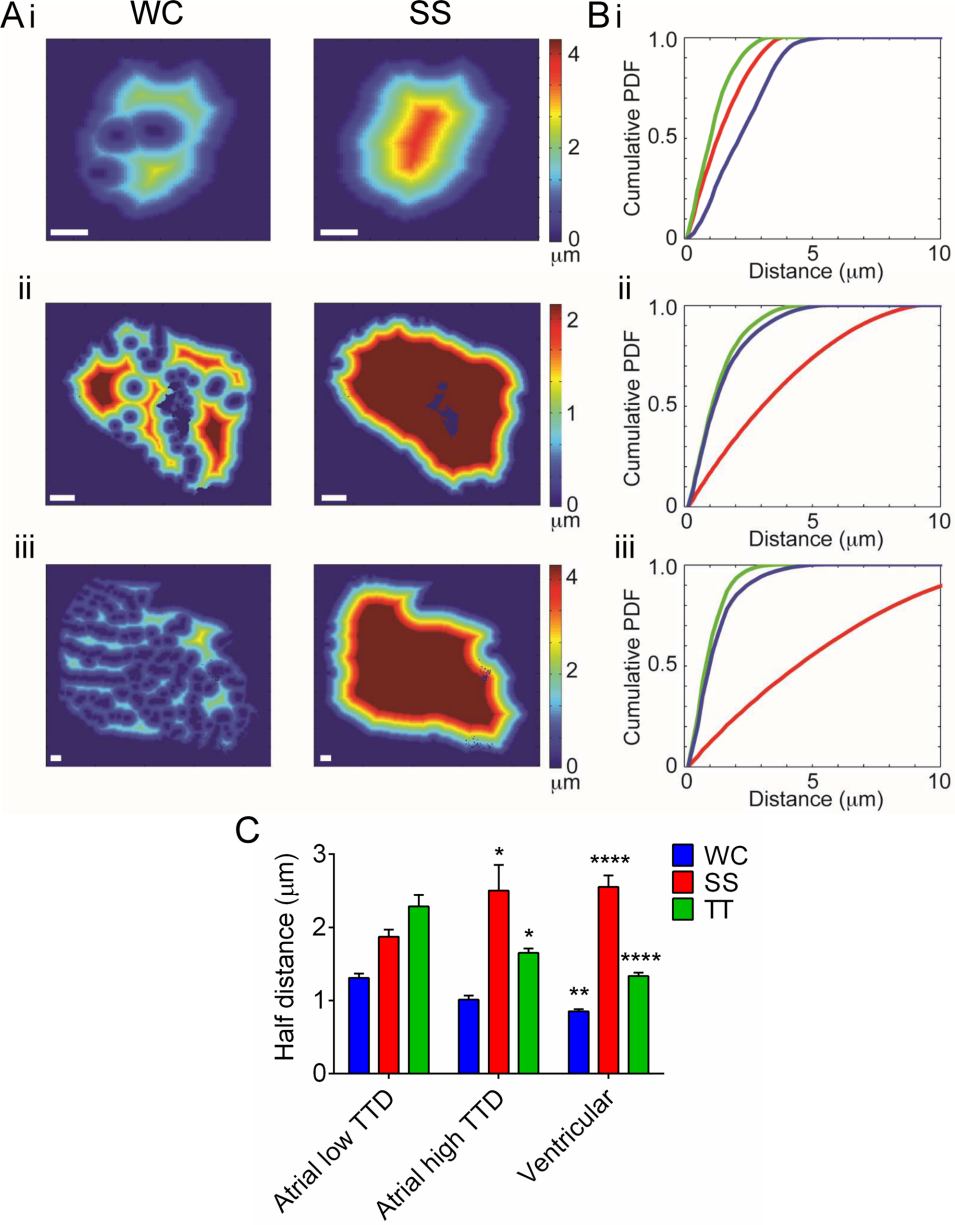
Distance maps in selected atrial and ventricular cells from tissue sections. A) Representative distance maps in (i) atrial cells with ‘low TTD’, (ii) atrial cells with ‘high TTD’ and (iii) ventricular cells. Scale bar represents 12 μm. Calibration bar shows distance to nearest membrane. B) Representative cumulative histograms for each cell type (blue–all membrane, red–surface membrane only, green–t-tubule membrane only). C) Mean half-distance to nearest membrane. * P<0.05, ** P<0.01, *** P<0.001 one-way ANOVA with Bonferroni post-test. Atrial cells with ‘low TTD’ *n* = 17, atrial cells with ‘high TTD’ *n* = 5, ventricular cells *n* = 24.

Distance maps were calculated for the whole cell membrane (WC), the t-tubule membrane alone (TT) and for the surface sarcolemma alone (SS) (Fig 6). Clear distinctions can be seen on the basis of this analysis between atrial cells with ‘low TTD’ and ventricular and atrial cells with ‘high TTD’ (Fig 6B). For example, the cumulative distribution of distance to the t-tubule membrane is close to that of the whole cell membrane in atrial cells with ‘high TTD’ and in ventricular cells but quite distinct in atrial cells with ‘low TTD’ (Fig 6B). Thus, it follows that in atrial cells with ‘low TTD’, the *HD*_*TT*_ was significantly greater than the half-distances to the WC (*HD*_*WC*_) and SS membranes (*HD*_*SS*_) whereas in cells with t-tubules, whether atrial or ventricular, *HD*_*TT*_ was significantly less than *HD*_*SS*_ but not significantly different to *HD*_*WC*_ (Fig 6C).

## Discussion

These data demonstrate considerable heterogeneity in the extent of the t-tubule network in both ventricular and atrial myocytes. The extent of the t-tubule network was strongly correlated with cell width. Our data show a high degree of heterogeneity between pig atrial cells in the extent of the t-tubule network area, a result similar to that recently reported elsewhere [32]. Moreover, the present study provides data from ventricular cells for comparison that also indicates a hitherto un-appreciated degree of heterogeneity in the t-tubule network amongst healthy ventricular cells. Our data imply that cell size is a key determinant of the extent of the t-tubule network in both atrial and ventricular cells.

In the present study, enzymatically isolated atrial myocytes showed sparse and irregular t-tubular staining. However, approximately 30% of pig atrial myocytes in tissue sections showed evidence of a more organized t-tubular network. Presumably, the sparsity of t-tubules from the enzymatically isolated atrial cells in the present study reflects lability of t-tubules and their loss during the enzymatic isolation procedure [44]. In any case, the finding of a t-tubular network in some atrial myocytes from the stained sections is in good agreement with a recent report by Frisk *et al*. suggesting that 28.5% of pig left atrial myocytes have a well-developed t-tubule network [32]. At first sight, this result appears to reflect the heterogeneity previously reported for the sheep atrium, although few small cells essentially lacking t-tubules were reported in those studies [28, 30].

Although in the present study t-tubule density (TTD) was significantly different between atrial and ventricular cells, both cell types showed a marked heterogeneity in t-tubule density and there was a considerable degree of overlap in TTD between the two groups of cells. Importantly, t-tubule density correlated significantly with short axis cell diameter, implicating cell size as a key factor in the development of the t-tubule network. Since all ventricular cells had a well-organized t-tubular network and had t-tubule density of >3%, the atrial cells were divided into two groups; those that had t-tubule density >3%, overlapping with ventricular cells, and those that had t-tubule density <3%. Pig atrial cells with high t-tubule density and pig ventricular cells were indistinguishable in terms of cell size, half-distance to t-tubule membrane and t-tubule density. On the other hand, the mean half-distances to t-tubule of pig atrial cells with low t-tubule density was consistent with the comparative lack of t-tubules in these cells. The degree of variation in t-tubule density and half-distance to t-tubule amongst the pig ventricular cells (e.g. Figs 5B and 6B) was unexpected given a previous report that in sheep ventricular cells half-distance to t-tubule was independent of cell size [28]. In the smaller pig ventricular cells of the present study, t-tubules were not present at each z-line location. Consistent with this observation, significant inhomogeneities in the localization of Ca^2+^ release have been reported in isolated pig ventricular myocytes [46]. It is unlikely that the heterogeneity in the t-tubular network was related to age or developmental stage as the pigs used were young adults and electrocardiogram recordings of the P wave in pigs of different ages have suggested that the electrophysiology of the atrial myocardium is fully developed by this stage [47–49].

**Fig 7:**
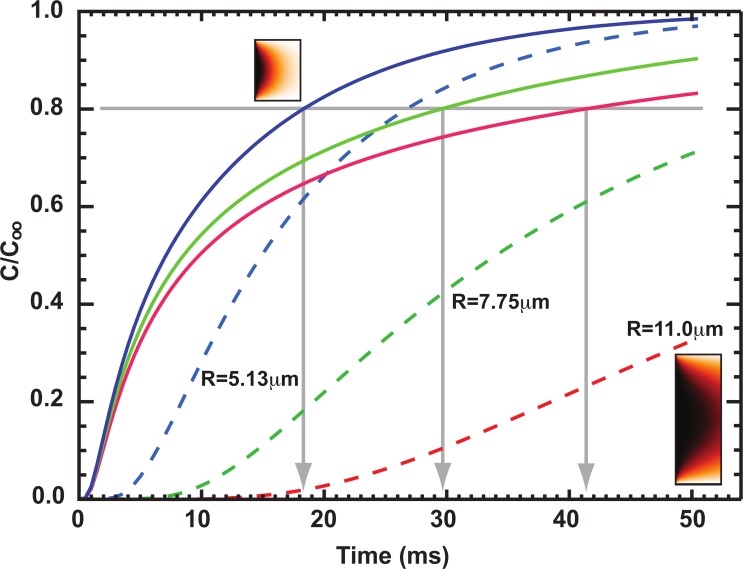
Relation between cell size and time to reach steady-state for Ca^2+^ release at the cell edge. The Ca^2+^ concentration, expressed as a fraction of the steady state value (C/C_∞_) at 2 μm from a site of release at the periphery of the cell (solid lines) and at the center of the cell (dashed lines) for cells of 5.13 μm (red), 7.75 μm (green) and 11.0 μm (blue) radius. Insets show simulated transverse line-scan images for changes in [Ca^2+^]_i_ with 5.13 μm (top left) and 11.0 μm (bottom right) radius.

Our data are consistent with cell size representing an important determining factor in t-tubule density. For example, while sheep atrial cells have been reported to show an extensive t-tubule network with less apparent heterogeneity than we and others have found in the pig atrium, some degree of variation in half-distance to t-tubule was reported and *HD*_*TT*_ correlated with cell width [28, 30, 32]. To demonstrate the requirement of larger cells for a t-tubule network, we modelled centripetal diffusion of Ca^2+^ from a release site at the cell periphery in cylindrical cells of different cross-sectional radius without t-tubules. Fig 7 shows the time course of change in [Ca^2+^]_i_ expressed as a proportion of the steady-state value (C/C_∞_) for cells of different size (cross-sectional radius) representative of the cell types examined in the present study. Change in C/C_∞_ at 2 μm from the release site (solid lines) and at the center of the cell (dashed lines) were plotted against time. As a consequence of the effects of diffusion, C/C_∞_ rose at the cell center with a considerable delay compared with 2 μm from the release site for all radii. However, the time to reach 80% C/C_∞_ at 2 μm distance (similar to the length of a sarcomere) was also highly dependent on cell radius. While in a cell with radius of 5.25 μm (representative of atrial cells with low TTD in the present study), C/C_∞_ reached 80% in ~18 ms (i.e. well within the half time of a normal calcium spark), in a cell with radius of 11.0 μm (representative of larger ventricular cells), 80% C/C_∞_ at 2 μm was reached in ~41 ms which is longer than the half time of a calcium spark. Therefore the contribution of t-tubules to spatially synchronous Ca^2+^ changes becomes even more critical as cell radius increases. It is also apparent (from the time taken for [Ca^2+^] to rise at the center and at 2 μm) that in small diameter cells (regardless of source), t-tubules would make a relatively smaller impact to the synchrony of [Ca^2+^] increase. It follows that the requirement for the existence of a t-tubule network to ensure synchronous Ca^2+^ release is the same for atrial and ventricular cells.

In summary, our data demonstrate considerable heterogeneity between pig cardiomyocytes in the extent of the t-tubule network. While the spatio-temporal properties of the Ca^2+^ transient in fluo-3-loaded isolated atrial cells were consistent with the relative absence of t-tubules from these cells [6], the loss of t-tubules in these cells makes more general conclusions about spatio-temporal properties of the Ca^2+^ transient uncertain. Resolving this uncertainty will require tissue level Ca^2+^ imaging, which was not possible in the present study. Nevertheless, it is likely that spatial non-uniformities in Ca transients will be reduced by the presence of a t-tubule network and this is strongly related to cell size. That there is such variation within the atria in the extent of the t-tubule network within the normal heart should sound a cautionary note in the interpretation of data from translational models of atrial (patho)physiology in larger species. On the other hand, the data also indicate that further studies are required to determine the role of t-tubules to normal atrial function and dysfunction.

### Limitations to the present study

In fixed tissue, in which the sarcolemma will have been permeabilized, nuclear core complexes and the Golgi apparatus will have been stained by WGA [39, 40]. Although staining of these structures has been removed digitally, the contribution of these structures to the image analysis cannot be entirely ruled out. However these structures occupy a small fraction of cell volume so their effect on the data analysis should be small.
